# Shape Memory Polymers as Functional Platforms for Dynamic Tissue Engineering and Regenerative Medical Devices

**DOI:** 10.3390/polym18182250

**Published:** 2026-09-15

**Authors:** Kyubae Lee

**Affiliations:** 1 Department of Biomedical Engineering, Gachon University, Seongnam 13120, Republic of Korea; kyubae@gachon.ac.kr; 2 Department of Biomedical Materials, Konyang University, Daejeon 35365, Republic of Korea

**Keywords:** shape memory polymers, functional polymers, tissue engineering, regenerative medicine, dynamic scaffolds, 4D printing, biodegradable polymers, stimuli-responsive biomaterials, minimally invasive implants

## Abstract

Shape memory polymers (SMPs) have emerged as a distinctive class of functional polymers for tissue engineering and regenerative medical devices because they couple programmable shape transformation with the biological, mechanical, and degradation requirements of regenerating tissue. Unlike conventional static scaffolds, SMP-based constructs can be delivered in compact temporary configurations, deployed under clinically relevant stimuli, and recovered into porous, anatomically conformal architectures that mechanically and biologically engage with host tissue. This review provides an engineering-oriented perspective on SMPs as functional polymers, emphasizing how shape recovery alone is insufficient and how regenerative performance emerges from coupled control of polymer chemistry, transition behavior, recovery force, fixity, degradation kinetics, and cytocompatibility. Material platforms ranging from biodegradable polyesters and polyurethanes to hydrogels, natural-polymer composites, and dynamic covalent networks are compared in terms of their ability to support tissue-specific functions including minimally invasive deployment, self-fitting, mechanical conditioning, and staged remodeling. The review further discusses 4D-printed patient-specific architectures and translational barriers, including sterilization, packaging, fatigue, manufacturing reproducibility, and regulatory validation. Together, these perspectives reframe SMPs from shape-recovery materials into integrated regenerative engineering platforms guiding the next generation of dynamic polymeric biomaterials.

## 1. Introduction

Tissue engineering has traditionally relied on scaffolds that provide structural support, spatial guidance, and biological cues for repair and regeneration, a paradigm established by Langer and Vacanti [1] and elaborated in broader surveys of biomaterials and scaffolds [2] and in scaffold-design frameworks for bone and cartilage [3]. Most conventional scaffolds are designed as static three-dimensional matrices with predetermined geometry, porosity, stiffness, and degradation behavior. Although these features support initial cell attachment, nutrient diffusion, and matrix deposition, tissue regeneration itself is not a static event. It proceeds through temporally evolving phases such as inflammation, cell recruitment, vascularization, matrix remodeling, mechanical maturation, and gradual replacement of the scaffold by newly formed tissue. Scaffolds whose structural and mechanical properties remain unchanged throughout this process therefore satisfy only a fraction of the dynamic requirements of complex tissue repair.

This mismatch between static scaffold design and dynamic regeneration has stimulated growing interest in functional polymers that respond to physical, chemical, or biological stimuli. Among these, shape memory polymers (SMPs) have emerged as particularly promising candidates because they can be programmed into a temporary configuration and subsequently recover a predefined permanent shape under a variety of stimuli, including heat [4], light [5], magnetic fields [6], electrical current [7], hydration [8], and pH change [9]. The biomedical potential of biodegradable, elastic SMPs was first demonstrated by Lendlein and Langer [10]; the underlying switching mechanisms and structure-property relationships were established in early reviews [11,12] and later expanded in a comprehensive survey of SMP structure, mechanism, functionality, and modeling [13]. This programmable behavior allows scaffolds and implants to be delivered in compact forms, deployed within confined anatomical spaces, and transformed into functional architectures at the target site. It directly addresses one of the longstanding challenges in tissue engineering, namely the geometrical conflict between minimally invasive delivery and effective tissue reconstruction [14,15].

From an engineering standpoint, SMPs are attractive because their functional behavior can be tuned across multiple length scales. Polymer chemistry, molecular architecture, crosslinking density, crystallinity, phase separation, composite formulation, and fabrication strategy all influence the transition temperature (*T_trans_*), the recovery ratio (*R_r_*), the shape fixity (*R_f_*), and the recovery force (*F_rec_*). Representative engineering analyses include the quantitative characterization of unconstrained recovery in SMP networks for cardiovascular use [16], the reprogramming of recovery and actuation behavior [17], and the coupling of these parameters with multifunctional drug release [18]. *T_trans_* dictates whether activation is feasible under physiologically and clinically acceptable conditions. *R_f_* governs whether the programmed temporary geometry survives sterilization, packaging, storage, and surgical handling. Recovery accuracy and force determine whether the scaffold restores its intended geometry and interacts safely with surrounding tissues. Coupled with degradation rate, cytocompatibility, bioactivity, and immune response, these parameters define whether an SMP construct is suitable for a given regenerative application.

Recent advances in polymer chemistry and additive manufacturing have considerably broadened this design space. Biodegradable shape memory networks based on polycaprolactone (PCL), poly(lactic acid) (PLA), poly(L-lactide-co-trimethylene carbonate) (PLLA-TMC), polyurethanes, and hydrogel-like systems combine shape programmability with tissue-compatible degradation, as illustrated by degradable drug-eluting networks [19], oligo(ε-caprolactone) AB-networks [20], and thermomechanical modeling of polyurethane-series SMPs [21], and more recently by 3D- and 4D-printed degradable scaffolds engineered to recover near physiological temperature [22,23]. Composite strategies have further extended functionality; examples include fast self-expandable biodegradable stents [24], stimuli-responsive SMP composites surveyed in dedicated reviews [25,26], and remote actuation by inductive heating of embedded magnetic nanoparticles [6]. Recent composite scaffolds that combine fast shape recovery with bioactive ceramic reinforcement further illustrate this direction [27]. In parallel, multimaterial 4D printing of SMP structures with tailorable behavior [28] builds on the broader concept of 4D printing introduced by Tibbits [29], with subsequent materials and applications surveyed elsewhere [30]. These developments position SMPs not merely as responsive polymers, but as integrated functional platforms for dynamic tissue engineering. These directions have been consolidated in recent comprehensive reviews of shape memory scaffolds for regenerative outcomes [31] and of SMP materials and their emerging biomedical applications [32].

Despite this momentum, the gap between proof-of-concept demonstrations and clinically translatable regenerative devices remains substantial. Many studies report impressive shape transformation under controlled laboratory conditions, but fewer address the full set of constraints required for medical-device-grade performance. Activation conditions must be safe for surrounding tissues and, in cell-laden systems, compatible with cell viability. *F_rec_* must support defect filling or scaffold deployment without injuring fragile tissues. Programmed shapes must remain stable through sterilization, packaging, transport, storage, and surgical handling. In biodegradable systems, mechanical integrity and shape-memory function must be maintained long enough to support tissue formation, while degradation products must remain non-toxic and non-inflammatory [33,34]. Material-level recovery tests alone cannot capture these requirements.

Accordingly, SMPs for tissue engineering should be evaluated not only as smart materials but as engineered regenerative systems. A polymer that recovers efficiently in a beaker may still fail clinically if its activation condition is impractical, its degradation profile is poorly controlled, its mechanical response is mismatched to the target tissue, or its programmed shape cannot survive standard medical-device processing. Conversely, well-engineered SMP systems can deliver functions that no static scaffold easily provides, including minimally invasive deployment, self-fitting behavior in irregular defects, dynamic mechanical conditioning, and patient-specific implantation.

**Figure 1:**
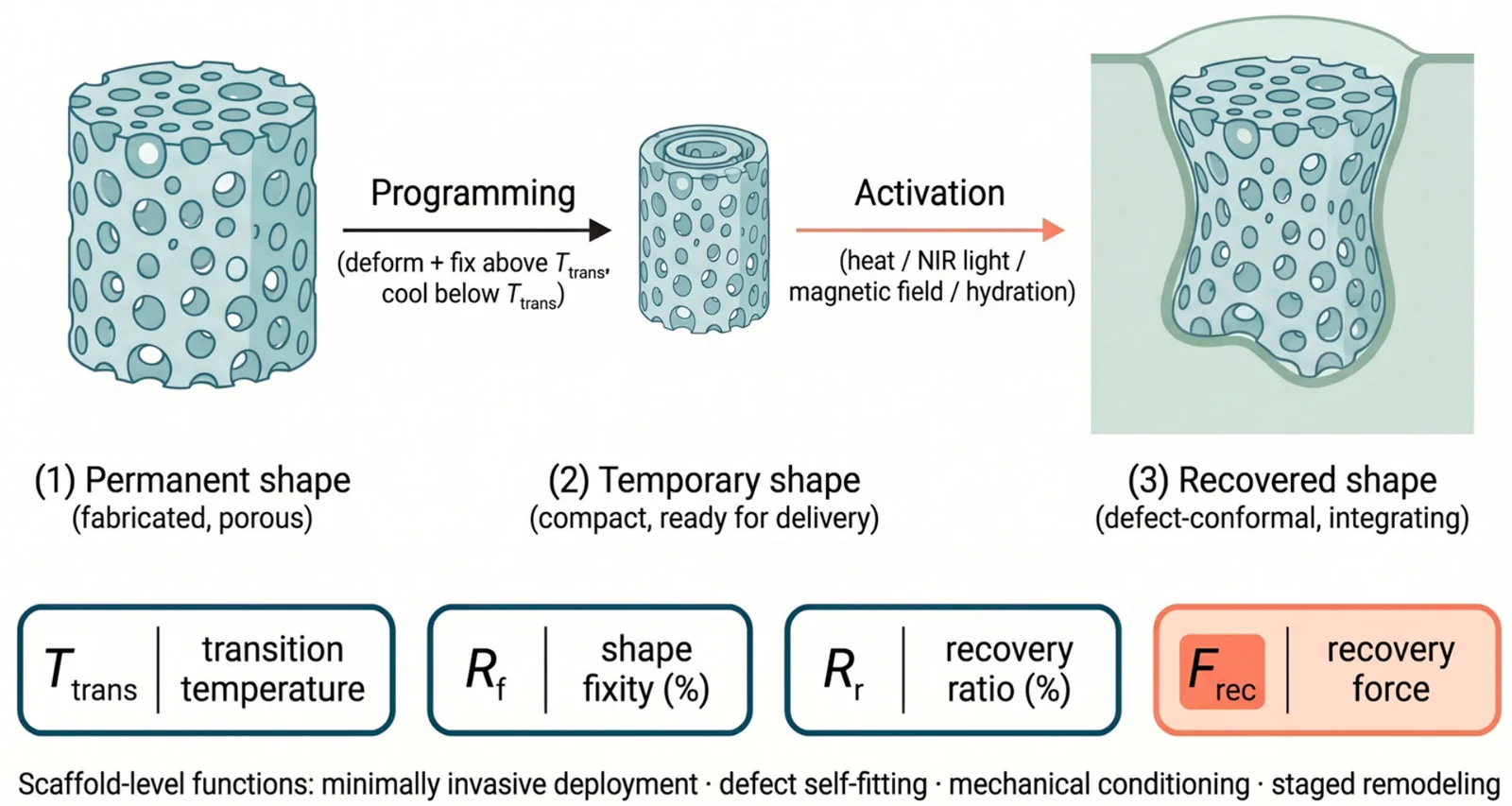
Conceptual scheme illustrating how an SMP scaffold is fabricated in its permanent shape, programmed into a compact temporary configuration for minimally invasive delivery, and recovered into a defect-filling architecture under physiologically compatible stimuli. The four core design parameters (*T_trans_*, *R_f_*, *R_r_*, and *F_rec_*) connect material-level behavior to scaffold-level functions such as deployment, self-fitting, mechanical conditioning, and staged remodeling.

This review takes an explicitly engineering-oriented perspective on SMPs as functional polymers for dynamic tissue engineering and regenerative medical devices. Rather than surveying SMPs as a broad class of smart materials, it focuses on the design requirements, material platforms, programmed scaffold functions, 4D-printed architectures, tissue-specific applications, and translational barriers that determine clinical relevance. By shifting attention from passive scaffold design to dynamic regenerative engineering, the review aims to clarify how SMPs can move beyond proof-of-concept actuation toward clinically meaningful platforms that integrate minimally invasive delivery, adaptive scaffold architecture, biological functionality, and translational feasibility (Figure 1).

## 2. Review Scope and Methodology

This article is structured as a critical, design-oriented narrative review rather than a systematic review or meta-analysis. Its purpose, scope, function, and intent are defined as follows.

The primary purpose is to reframe SMPs from materials defined by shape recovery into integrated regenerative engineering platforms, and to clarify how regenerative performance emerges from the coupled control of polymer chemistry, transition behavior, recovery force, shape fixity, degradation kinetics, and cytocompatibility rather than from shape recovery alone.

The review covers thermally and remotely activated SMPs used as scaffolds or implantable devices for tissue engineering and regenerative medicine. It encompasses (i) device- and tissue-level engineering requirements, (ii) material platforms, including biodegradable polyesters, segmented polyurethanes, hydrogel-like and water-responsive systems, natural-polymer and bioactive composites, and dynamic covalent networks, (iii) programmed scaffold functions, (iv) 4D-printed patient-specific architectures, (v) tissue-specific applications across bone, cartilage, vascular, cardiac, neural, and wound-healing targets, and (vi) translational barriers. Shape memory alloys, non-biomedical SMP applications such as aerospace, textiles, and soft robotics, and purely actuation-focused studies without a regenerative context were treated as outside the scope and were cited only where required for mechanistic context.

The review functions as a comparative and integrative synthesis. Rather than cataloguing all reported SMP systems, it compares representative platforms against tissue-specific requirements and connects material-level parameters (*T_trans_*, *R_f_*, *R_r_*, and *F_rec_*) to scaffold- and device-level functions and translational constraints.

The intended outcome is a design framework that helps researchers and engineers select and engineer SMP systems for defined tissue targets, and that highlights translational gaps, namely sterilization stability, programmed-shape shelf life, mechanical fatigue, manufacturing reproducibility, and regulatory validation, that must be addressed for clinical use.

Relevant literature was identified through Web of Science, Scopus, PubMed, and Google Scholar using combinations of search terms including shape memory polymer, tissue engineering, regenerative medicine, scaffold, 4D printing, biodegradable polymer, and tissue-specific keywords. Priority was given to peer-reviewed studies reporting shape-memory behavior together with biological, mechanical, or degradation data relevant to regenerative devices. Foundational works establishing SMP thermomechanical principles were retained for context, whereas emphasis was placed on developments from the last five years to reflect current trends. Studies without a tissue-engineering or regenerative-device context, as well as non-peer-reviewed sources, were excluded. Beyond the qualitative growth of this field, its scale can be quantified through recent bibliometric work. A CiteSpace analysis of the Web of Science Core Collection identified 926 publications on shape memory polymers for tissue engineering, biomedical applications, and regeneration published between 2000 and mid-2024, with a marked acceleration in the most recent years [35]. This review is positioned against that rapidly expanding body of work, and the emphasis on developments from the last five years reflects the period in which most of these studies have appeared.

## 3. Engineering Requirements for Shape Memory Polymers in Tissue Engineering

For SMPs to function as regenerative scaffolds or implantable tissue engineering platforms, shape recovery alone is insufficient. Their performance must be evaluated against device-level and tissue-level requirements, including geometric recovery, activation safety, mechanical compatibility, degradation behavior, biological response, and process stability. These parameters jointly determine whether an SMP-based scaffold can be delivered, deployed, integrated, and maintained in a regenerative environment.

### 3.1. Shape Fixity, Recovery Ratio, and Geometric Accuracy

*R_f_* and *R_r_* are the most fundamental performance indicators. *R_f_* describes the ability of a programmed scaffold to maintain its temporary geometry before activation, whereas *R_r_* indicates the extent to which the material returns to its predefined permanent shape after stimulation [10,17]. In tissue engineering, both parameters connect directly to scaffold delivery and defect reconstruction. A scaffold with poor *R_f_* may partially recover during storage, sterilization, or implantation, leading to unpredictable handling and inaccurate placement; incomplete *R_r_* may prevent the scaffold from filling an irregular defect or restoring its intended porous architecture.

Geometric accuracy is particularly important for patient-specific defects. Bone void fillers, osteochondral scaffolds, vascular conduits, and wound matrices typically require close anatomical conformity to ensure stable contact with host tissue. Even materials with high *R_r_* in simplified laboratory tests may underperform in physiological environments due to friction, confinement, hydration, tissue resistance, or partial degradation [36]. SMP scaffolds should therefore be evaluated not only by recovery percentage but also by dimensional accuracy, architectural restoration, and functional fit within defect-relevant models.

### 3.2. Transition Temperature and Safe Activation Window

Transition temperature is critical because it defines when and how shape recovery is triggered. Thermally activated SMPs typically use either glass transition temperature (*T_g_*) or melting transition temperature (*T_m_*) as the switching point [10,17]. For regenerative applications, this transition should be positioned within a clinically acceptable activation window. Activation near body temperature enables passive deployment after implantation, while slightly higher transitions allow controlled activation using warmed saline, photothermal heating, or magnetic induction.

Activation temperature must be balanced with tissue safety. Excessive heating may damage surrounding tissues, denature proteins, impair cell viability, or induce inflammation. This is especially important for cell-laden scaffolds, soft-tissue applications, neural interfaces, and cardiac patches [4,37]. Conversely, transition temperatures too close to room temperature can compromise temporary-shape stability during storage and handling. *T_trans_* should therefore be selected according to the target tissue, implantation route, activation method, and intended surgical workflow.

### 3.3. Recovery Force, Actuation Speed, and Tissue Compatibility

*F_rec_* determines whether an SMP scaffold can perform mechanical work during deployment, including expanding a scaffold, opening a lumen, filling a defect, maintaining tissue contact, or stabilizing an implant [10,38]. Sufficient *F_rec_* can improve defect filling and initial fixation in bone or osteochondral defects, but excessive force in vascular, cardiac, neural, or soft tissues may cause compression, irritation, tearing, or impaired tissue function.

Actuation speed is equally important. Rapid recovery is advantageous when immediate deployment or fixation is required, but abrupt expansion may damage delicate tissues. Slower, gradual recovery improves conformability, reduces local stress concentration, and supports adaptation to the surrounding microenvironment. *F_rec_* and actuation speed should therefore be co-designed with tissue stiffness, anatomical confinement, and the intended mode of implantation. The ideal SMP scaffold provides enough mechanical action to achieve functional deployment while minimizing trauma to the host tissue.

### 3.4. Mechanical Stability, Degradation, and Regenerative Time Window

Mechanical stability is essential because regenerative scaffolds must maintain function during the early and intermediate phases of tissue repair. Depending on the target tissue, SMP scaffolds may need to resist compression, bending, cyclic loading, swelling, or fatigue. Bone scaffolds require high stiffness and structural integrity, whereas cardiac, neural, or soft-tissue scaffolds require compliance and flexibility. Mechanical properties should therefore be matched to the target tissue rather than maximized without context [36,39]. Representative mechanical and shape-memory properties of SMP systems reported in the literature, together with the methods used to obtain them, are summarized for comparison (Table 1).

For biodegradable SMPs, degradation must be coordinated with the regenerative time window. The scaffold should retain sufficient mechanical strength and shape-memory function until newly formed tissue can provide structural support [19,43]. Premature degradation may cause loss of scaffold integrity, collapse of porous architecture, or failure of defect stabilization, while excessively slow degradation prolongs foreign-body exposure and interferes with remodeling. Degradation products must also be non-toxic, non-inflammatory, and compatible with local tissue homeostasis. Degradation rate, mechanical retention, and tissue formation should therefore be evaluated as interconnected parameters.

From an engineering standpoint, these parameters become useful only when their absolute values are matched to the target tissue rather than optimized in isolation. Representative SMP systems achieve shape-fixity and recovery ratios above roughly 90% (Table 1), but the mechanically decisive quantities for a scaffold are the recovery stress generated during deployment and the modulus in the recovered state. Across reported biomedical SMP systems, these span a wide window, from recovery stresses well below 1 MPa and moduli on the order of 1 MPa for elastomeric and hydrogel-like networks to several MPa and the order of 1 GPa for semicrystalline or ceramic-reinforced systems (Table 1). Mapping these values onto tissue requirements clarifies why no single material is universal: outputs at the upper end of this range suit the filling and stabilization of stiff bone voids (E ≈ 0.1 to 10 GPa), whereas the same mechanical action in vascular or neural tissue (E in the kPa to low-MPa range) would risk over-expansion or compression injury. The engineering objective is therefore to select a material whose quantitative property window overlaps that of the target tissue while keeping the activation temperature within the safe physiological range, a constraint that the switching mechanism alone does not capture.

### 3.5. Cytocompatibility, Immune Response, and Tissue Integration

Biological compatibility is a central requirement. The scaffold surface influences protein adsorption, cell adhesion, proliferation, differentiation, macrophage polarization, and vascular ingrowth. Polymer chemistry, surface wettability, roughness, degradation products, residual monomers, and incorporated fillers can all shape the host response [18,44]. Cytocompatibility should be assessed in relation to the intended tissue and cell type rather than treated as a generic property.

Immune response is also critical. A moderate, transient inflammatory response can support tissue repair, whereas chronic inflammation can lead to fibrotic encapsulation, poor integration, or implant failure. SMP composites containing nanoparticles, conductive fillers, ceramics, or bioactive molecules require additional evaluation because these components may alter oxidative stress, macrophage behavior, or local microenvironmental conditions [7,45]. The goal is not merely to avoid cytotoxicity but to construct a material-tissue interface that actively supports cell recruitment, vascularization, matrix deposition, and functional remodeling.

Taken together, these requirements emphasize that SMPs should be evaluated as regenerative systems rather than isolated smart materials. *R_f_*, recovery behavior, activation safety, mechanical performance, degradation kinetics, and biological response are interdependent, and the optimal balance among them depends on the target tissue, scaffold architecture, implantation route, and clinical application. This design-oriented perspective sets the stage for the material platforms and functionalization strategies discussed in the next section.

## 4. Material Platforms and Functionalization Strategies

**Figure 2:**
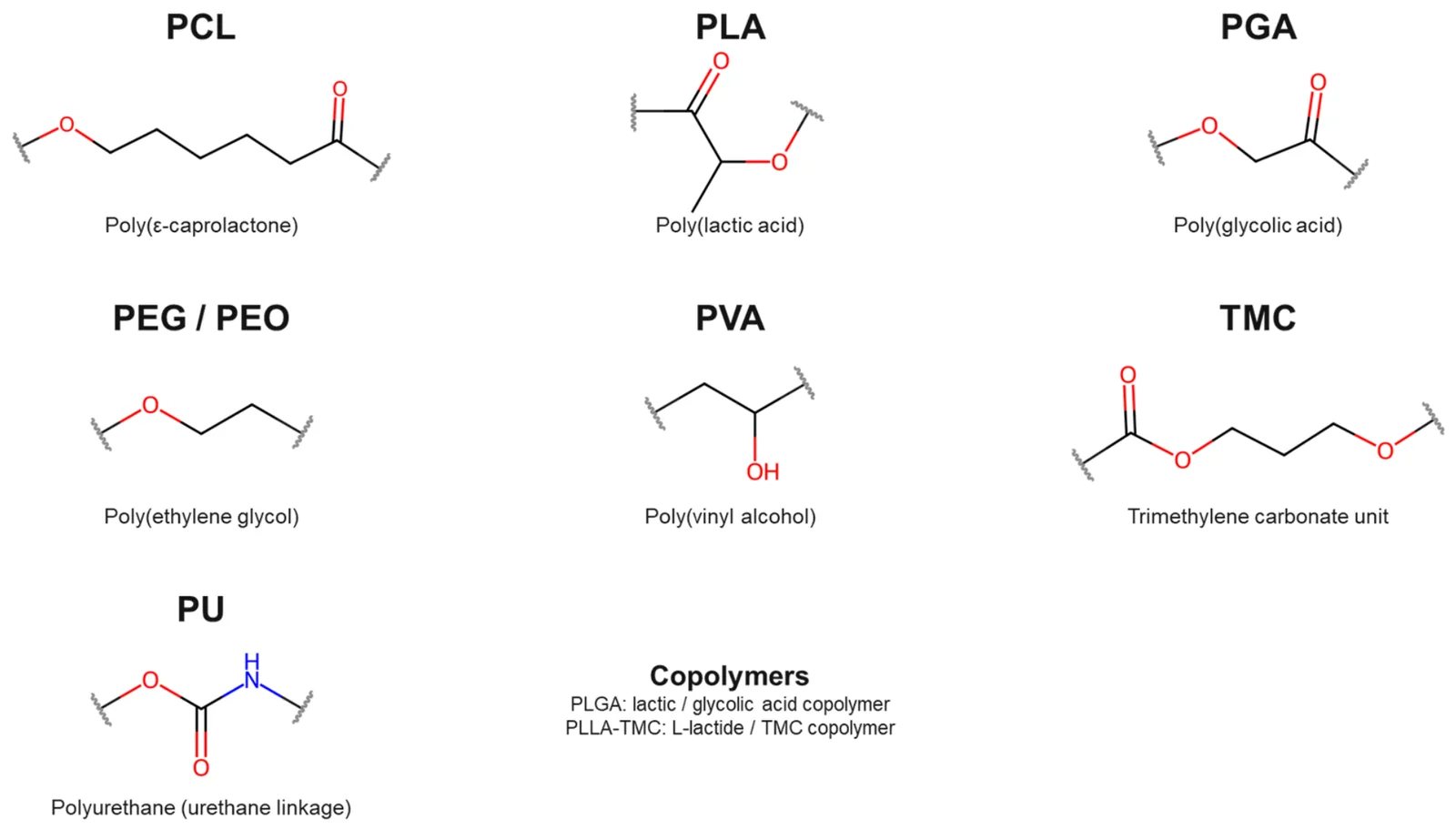
Representative repeat-unit structures of the principal polymer chemistries used in shape memory polymers for tissue engineering. Wavy bonds indicate the attachment points at which each repeat unit connects along the polymer chain. PCL, PLA, and poly(glycolic acid) (PGA) are the principal biodegradable polyesters, and poly(lactic-co-glycolic acid) (PLGA) is a copolymer of the lactic- and glycolic-acid units. PEG, also termed poly(ethylene oxide) (PEO), and poly(vinyl alcohol) (PVA) provide hydrophilic, hydrogel-forming segments. The trimethylene carbonate (TMC) unit appears in PLLA-TMC networks, and the urethane linkage shown is the characteristic bond of shape memory polyurethane (PU).

Selection of an appropriate SMP platform is a decisive step in designing dynamic scaffolds and regenerative medical devices. The principal polymer chemistries used as SMPs and their repeat-unit structures are summarized (Figure 2). Different polymer systems provide distinct advantages in transition behavior, mechanical strength, degradation rate, elasticity, processability, and biological compatibility (Table 2). Material selection should therefore be guided by the target tissue, implantation route, required recovery behavior, and expected regenerative time window. SMPs are chosen not only for shape recovery but for their capacity to support deployment, tissue integration, mechanical adaptation, and functional regeneration.

### 4.1. PCL-, PLA-, and Polyester-Based Biodegradable SMPs

Polyester-based SMPs, particularly PCL-containing systems, are among the most widely investigated platforms because PCL combines biodegradability, a melting transition close to body temperature, processability, and compatibility with porous scaffold fabrication, building on early oligo(ε-caprolactone) shape-memory networks [19,20] and broader assessments of biodegradable biomaterials [43]. Neat PCL melts at roughly 60 °C, so for activation near body temperature the switching transition is engineered downward by copolymerization, blending with lower-melting or amorphous comonomers, control of molecular weight and crosslink density, or composite formation [41]. The high crystallinity that sharpens this transition also slows hydrolytic degradation, which often extends over one to three years, so that the scaffold can provide prolonged mechanical support but may outlast the regenerative window unless degradation is deliberately accelerated. These features make PCL-based SMPs suitable for minimally invasive scaffold delivery, self-expanding structures, and defect-adaptive implants. The foundational thermomechanical behavior underlying many subsequent biomedical SMP designs was established by these AB-polymer networks of Lendlein and colleagues [10,20].

PLA, PLGA, and related polyester systems offer additional options. Compared with PCL, these polymers generally provide higher stiffness and faster degradation, which can be advantageous for applications requiring temporary mechanical support and gradual replacement by newly formed tissue. The glass transition of PLA lies near 55 to 65 °C and depends on stereochemistry, with semicrystalline poly(L-lactide) (PLLA) and amorphous poly(D,L-lactide) (PDLLA) behaving differently, while the degradation rate of PLGA can be tuned from weeks to months through the lactide-to-glycolide ratio. However, bulk erosion releases acidic oligomers that can lower local pH and provoke inflammation, so acidic by-products and rapid loss of mechanical integrity must be carefully managed, often by buffering with bioceramic fillers or by blending with slower-degrading components [21,43]. Because crystallinity governs the melting transition and the degradation rate simultaneously, these two properties cannot be tuned entirely independently. Polyester-based SMPs are therefore particularly relevant for bone, cartilage, osteochondral, and selected soft-tissue applications where scaffold degradation should be synchronized with tissue formation.

### 4.2. Polyurethane-Based SMPs for Elastic and Soft-Tissue Applications

Shape memory polyurethanes (SMPUs) are versatile because their segmented architecture allows independent tuning of hard and soft domains. Hard segments, typically formed from a diisocyanate and a chain extender, create physical netpoints through hydrogen bonding and microphase separation that fix the permanent shape and contribute mechanical strength, whereas soft segments such as a PCL-diol, PEG, or polycarbonate-diol provide the reversible switching phase whose glass or melting transition sets the recovery temperature [17,21]. The degree of microphase separation governs shape fixity, recovery stress, and recoverable strain, which for SMPUs can exceed several hundred percent. This modular design provides control over elasticity, recovery temperature, *F_rec_*, and elongation, making SMPUs useful for soft and mechanically dynamic tissues.

In regenerative medicine, SMPUs are particularly attractive for vascular grafts, cardiac patches, wound dressings, and soft-tissue scaffolds, where elastic recovery improves tissue contact and reduces mechanical mismatch [37,46]. However, they require careful evaluation of degradation products, long-term stability, and inflammatory response. The choice of diisocyanate is decisive for safety, because aromatic diisocyanates such as methylene diphenyl diisocyanate (MDI) and toluene diisocyanate (TDI) can release toxic aromatic diamines on hydrolysis, whereas aliphatic or lysine-derived diisocyanates yield more benign degradation products and are generally preferred for implantable devices. For biodegradable SMPUs, the balance among elasticity, degradation rate, and biocompatibility is critical: excessively slow degradation may limit tissue remodeling, while rapid degradation may compromise mechanical support before regeneration is complete.

### 4.3. Hydrogel-Like and Water-Responsive SMP Systems

Hydrogel-like and water-responsive SMPs provide a biologically favorable environment owing to their high-water content, softness, and similarity to native extracellular matrix (ECM) [5,47]. These materials are particularly relevant for cell-laden scaffolds, injectable systems, and soft-tissue engineering. In these systems, the temporary shape is fixed by a physical network, such as vitrified or crystalline domains or reversible associations including poly(vinyl alcohol) crystallites and multiple hydrogen-bonding motifs, and recovery is triggered when water plasticizes the switching domains and lowers *T_trans_* toward body temperature, enabling shape recovery under mild conditions that is useful when high-temperature activation is undesirable. This water-driven mechanism was demonstrated for polyurethane systems in which sequential water uptake produced programmable recovery [8].

Their major advantage is the combination of shape programmability with cell compatibility and molecular transport. Hydrated networks support nutrient diffusion, waste removal, and cell infiltration, and can be functionalized with bioactive ligands, growth factors, peptides, or degradable linkages to drive tissue-specific regeneration. Their modulus, however, typically lies in the kilopascal to low-megapascal range, far below that of load-bearing tissue, and equilibrium swelling can change scaffold dimensions substantially, which restricts load-bearing applications. Hydrogel-like SMPs are therefore frequently combined with reinforcing networks, nanofillers, or interpenetrating polymer structures to improve structural stability while maintaining biological functionality.

### 4.4. Natural-Polymer-Containing and Bioactive SMP Composites

A recurring theme across these platforms is that the principal limitations of neat (unfilled) SMPs are most effectively addressed through composite formulation [48]. Single-component SMPs typically present one or more shortcomings for a given tissue. Semicrystalline polyesters such as PCL offer favorable processability and degradation but limited stiffness and no intrinsic osteoconductivity. Hydrogel-like and elastomeric SMPs provide cell-friendly, compliant environments but cannot bear significant load. Most thermally activated SMPs require direct, bulk heating that is impractical inside the body. Composite counterparts overcome these drawbacks selectively. Reinforcement with bioactive ceramics such as HA, CaP, or mesoporous bioactive glass simultaneously raises stiffness and confers osteoconductivity [40,49]. A recent shape-memory poly(L-lactide-co-trimethylene carbonate) scaffold loaded with mesoporous bioactive glass, for instance, reached a compressive strength of about 66 MPa at 30% filler while retaining thermoresponsive intraoperative molding and promoting osteogenic differentiation [27]. Blending with natural polymers restores the cell adhesion and enzymatic degradability that synthetic backbones lack. Magnetic and conductive fillers convert otherwise passive, insulating networks into remotely and electrically addressable systems. Inductive heating of embedded magnetic nanoparticles enables wireless actuation [6,45], superparamagnetic iron oxide additionally promotes osteogenesis [42], and carbon-nanotube-loaded polyurethanes provide electroactive shape recovery suited to cardiac and neural interfaces [7,50]. Architectural composites can also tune the transition itself, as in star-shaped PCL networks that shift the melting transition into a clinically useful range while preserving shape-memory fidelity [41]. In each case, the composite is engineered to supply a property the neat polymer lacks, which is why composite SMPs, rather than single-component systems, increasingly dominate tissue-specific scaffold design.

Natural polymers such as collagen, gelatin, chitosan, hyaluronic acid, alginate, and silk fibroin can be incorporated into SMP systems to enhance cell adhesion, matrix deposition, enzymatic degradability, and tissue integration [18,44]. Their inclusion is particularly useful when synthetic SMPs provide desirable mechanical or shape-memory behavior but lack sufficient biological cues.

Because incorporated fillers also alter *T_trans_*, stiffness, degradation, and immune response, a bioactive composite SMP must be evaluated as an integrated material system rather than a simple combination of polymer and additive.

### 4.5. Dynamic Covalent Networks and Reprocessable SMPs

Dynamic covalent networks, including vitrimer-like SMPs, have recently attracted attention because they offer reprocessability, stress relaxation, self-healing potential, and structural adaptability [51,52]. Unlike permanently crosslinked thermosets, these systems contain reversible bonds, such as transesterification, imine, and disulfide bonds, that can exchange under specific conditions while preserving network integrity. The nature of the exchange determines the behavior: associative networks such as transesterification-based vitrimers maintain a constant crosslink density and show Arrhenius-like viscosity above a topology-freezing temperature, whereas dissociative networks based on imine or Diels-Alder chemistry transiently break bonds in order to flow. This bond exchange allows a programmed permanent shape to be rewritten, scaffolds to be welded or reshaped, and damage to be healed, enabling more sophisticated programming of scaffold architecture.

For tissue engineering, dynamic covalent SMPs may offer benefits in personalized scaffold fabrication and post-processing, including improved defect matching, shape reprogramming, and damage tolerance. Their biomedical use, however, remains at an early stage. Many exchange reactions require catalysts, such as transesterification catalysts, or elevated temperatures, and the long-term hydrolytic stability of the dynamic linkages under physiological conditions remains uncertain. Open questions therefore include the stability of dynamic bonds under physiological conditions, the safety of bond-exchange chemistry, long-term mechanical reliability, and compatibility with sterilization and degradation requirements.

Overall, material platforms should be selected by functional need rather than by polymer class alone. Polyester-based SMPs offer biodegradable structural support; SMPUs provide elasticity and soft-tissue compatibility; hydrogel-like SMPs supply hydrated, cell-friendly environments; natural-polymer composites enhance biological interactions; and dynamic covalent networks introduce reconfigurability and repairability. The next challenge is translating these material properties into programmed scaffold functions that directly support tissue regeneration.

## 5. Programming Dynamic Scaffold Functions

**Figure 3:**
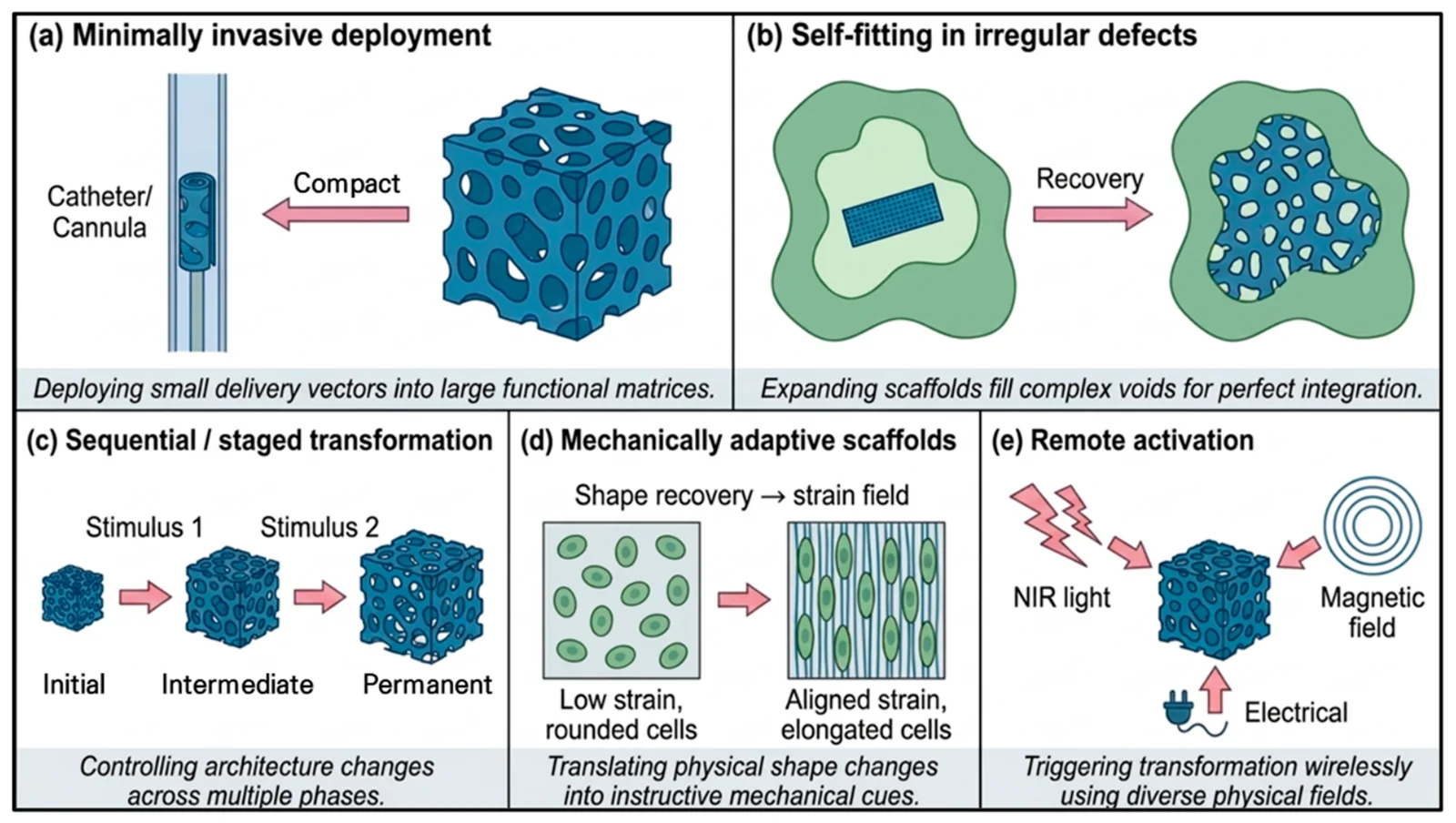
Programmed scaffold functions enabled by SMP design. (**a**) Minimally invasive deployment: a large functional scaffold is programmed into a compact temporary form that fits within the narrow lumen of a catheter or cannula, enabling delivery through small surgical accesses before recovery to its expanded geometry at the target site. (**b**) Self-fitting behavior, in which a programmed temporary shape recovers within an irregular tissue defect and conforms to the surrounding boundaries. (**c**) Sequential or staged transformation through multiple temporary configurations (Initial → intermediate → permanent) triggered by distinct stimuli, enabling architecture changes across multiple phases. (**d**) Mechanically adaptive scaffolds, in which shape-memory-actuated change in the underlying fiber alignment converts a low-strain substrate (rounded cells) into an aligned-strain substrate (elongated, oriented cells), translating physical shape change into instructive mechanical cues for adherent cells. (**e**) Remote activation by near-infrared (NIR) light, magnetic field, or electrical stimulus enables wireless triggering of shape recovery without direct mechanical access.

The principal advantage of SMPs in tissue engineering is not simply that they change shape, but that programmed deformation can be translated into scaffold-level functions, including minimally invasive delivery, self-fitting behavior, staged structural transformation, mechanical adaptation, and remote activation (Figure 3). Such functions allow scaffolds to interact with the defect environment in a time-dependent and tissue-specific manner that static scaffolds cannot match.

### 5.1. Minimally Invasive Deployment and Self-Expansion

Minimally invasive deployment is one of the most clinically relevant SMP-enabled functions. A scaffold can be compressed, folded, or rolled into a compact temporary shape before implantation and recover its permanent geometry at the target site, as shown for biodegradable SMP systems [10,19] and for self-deploying scaffolds [53]. Self-expanding SMP scaffolds can reduce surgical invasiveness while preserving the structural complexity required for regeneration. The deployment process must, however, be controlled: excessive *F_rec_* or rapid expansion may damage surrounding tissues, whereas incomplete recovery may compromise defect filling and mechanical stability.

### 5.2. Self-Fitting Behavior for Irregular Tissue Defects

Many tissue defects are irregular and patient-specific, making prefabricated static scaffolds suboptimal. Self-fitting SMP scaffolds address this by recovering within the defect space and conforming to surrounding tissue boundaries [40,53]. This is especially relevant for bone voids, osteochondral defects, craniofacial defects, wound beds, and complex soft-tissue defects, where improved geometric matching enhances initial fixation, reduces dead space, and supports more uniform tissue infiltration. The challenge is balancing recovery accuracy and *F_rec_* against defect confinement so that the scaffold expands enough to conform without producing local pressure damage.

### 5.3. Sequential Shape Change and Staged Tissue Repair

Tissue regeneration unfolds in stages, and a single fixed scaffold architecture is rarely optimal throughout. SMPs can be designed for sequential or staged shape changes via multi-phase networks, layered structures, spatially patterned transition temperatures, or multi-stimuli-responsive designs [30,42]. For example, an SMP scaffold may first expand to fill a defect and later undergo secondary deformation to improve mechanical support, open internal channels, or alter local porosity. Although technically demanding, such systems provide a useful framework for designing scaffolds that better reflect the temporal nature of tissue repair.

### 5.4. Mechanically Adaptive Scaffolds for Cell Conditioning

Mechanical cues strongly influence cell adhesion, proliferation, differentiation, migration, and matrix production. SMPs contribute to mechanobiology-guided tissue engineering by enabling time-dependent changes in scaffold stiffness, strain distribution, pore geometry, or local stress, modulating the cellular microenvironment, consistent with the established sensitivity of cells to substrate stiffness [36,39] and with shape-memory-actuated changes in fiber alignment that direct stem-cell morphology [54]. This is particularly relevant for mechanically responsive tissues such as bone, cartilage, tendon, cardiac tissue, and muscle. Mechanical adaptation must, however, be carefully programmed: uncontrolled deformation may disrupt cell attachment or matrix formation, whereas excessive stiffness may impair tissue remodeling.

### 5.5. Remote Activation Using Light, Magnetic, or Electrical Stimuli

Remote activation expands SMP utility by allowing shape change to be triggered after implantation without direct mechanical manipulation. Light-responsive systems offer spatially controlled activation in superficial or endoscopically accessible sites; magnetic activation enables deeper tissue triggering through inductive heating of embedded magnetic nanoparticles [6,45], and electrically responsive SMPs integrated with conductive fillers, microheaters, or bioelectronic components allow localized, controllable actuation through electroactive composites such as those incorporating carbon nanotubes [7]. Each modality, however, imposes specific safety and design constraints. Light suffers from limited tissue penetration and photothermal damage risk; magnetic systems require careful control of nanoparticle loading and heating efficiency; and electrical activation requires stable conductive networks and insulation from surrounding tissue. Remote activation should therefore be considered as part of the overall scaffold and device design rather than as a standalone triggering method.

Together, these programmed functions form the key bridge between material-level shape memory behavior and tissue-level regenerative performance. Their impact is amplified when combined with advanced fabrication, particularly 4D printing, which allows spatial control over scaffold architecture, deformation, and patient-specific geometry.

## 6. 4D Printing and Patient-Specific Regenerative Architectures

**Figure 4:**
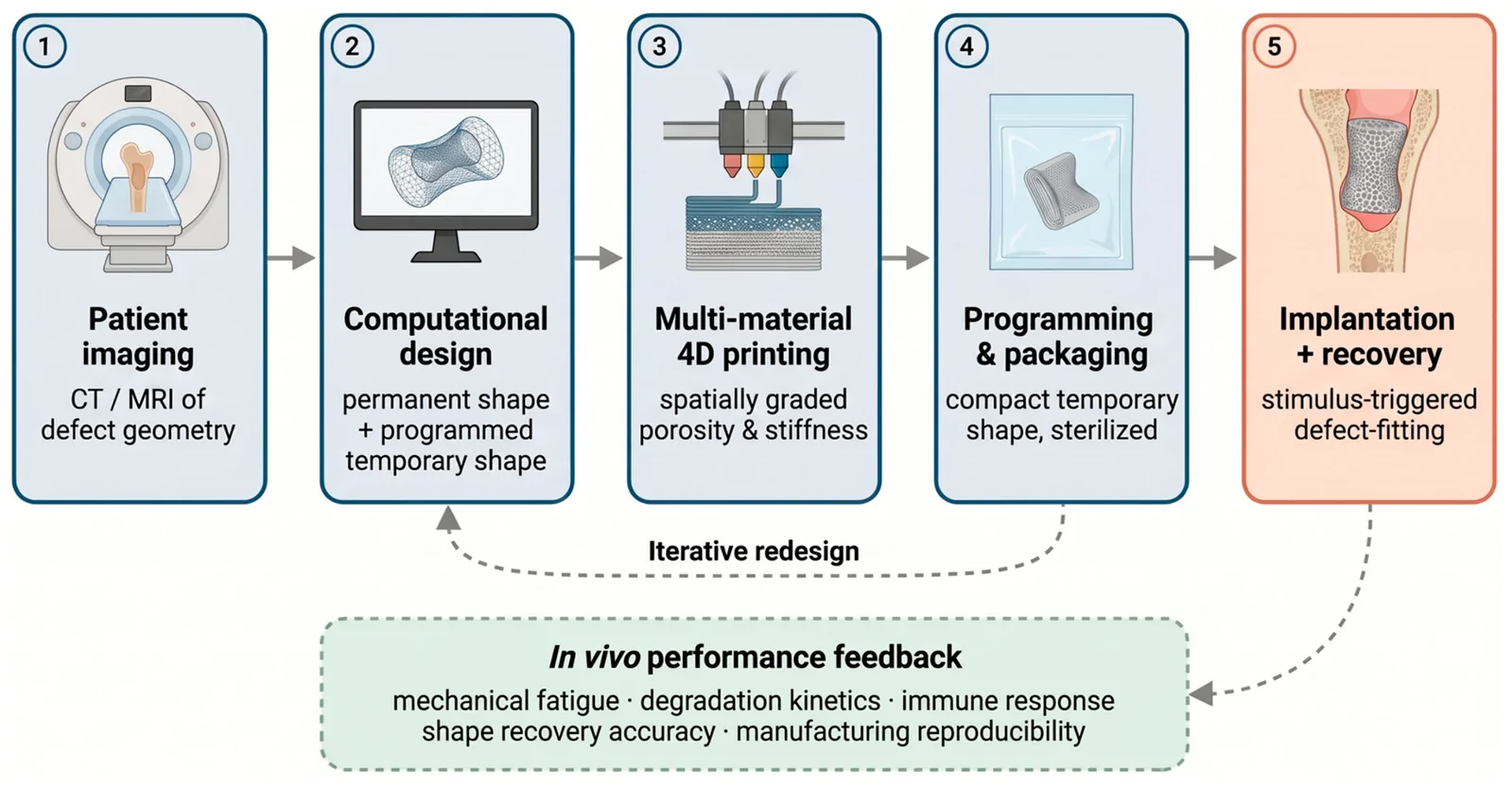
4D-printed patient-specific SMP scaffold workflow. (**1**) Patient imaging by computed tomography (CT) or magnetic resonance imaging (MRI) defines the geometry of the target defect. (**2**) This geometry informs computational design of both the permanent scaffold shape and its programmed temporary shape. (**3**) Multi-material 4D printing translates this design into a construct with spatially graded porosity and stiffness. (**4**) The construct is then programmed into a compact temporary configuration and sterilized for storage. (**5**) After implantation, the construct undergoes stimulus-triggered recovery to achieve defect-conformal deployment. In vivo performance feedback, including mechanical fatigue, degradation kinetics, immune response, shape recovery accuracy, and manufacturing reproducibility, drives iterative redesign at the material and device levels.

Additive manufacturing has become indispensable in tissue engineering because it enables scaffolds with controlled geometry, porosity, and patient-specific dimensions. When combined with SMPs, this approach extends from static three-dimensional fabrication to 4D printing, in which printed structures change shape or function over time in response to external or physiological stimuli, as demonstrated in early 4D printing studies [28,29] and extended to 4D bioprinting of stimuli-responsive materials [55]. 4D-printed shape memory biomedical devices, spanning bone scaffolds and vascular and tracheal stents, have recently been reviewed [56]. For regenerative medicine, 4D printing offers a powerful strategy to integrate anatomical customization, programmed deformation, and dynamic scaffold function within a single platform (Figure 4).

### 6.1. From 3D-Printed Scaffolds to 4D-Printed Regenerative Systems

Conventional 3D printing controls scaffold architecture, including pore size, interconnectivity, external shape, and internal channels. These features are critical for cell infiltration, nutrient transport, vascularization, and mechanical support. Yet most 3D-printed scaffolds remain structurally fixed after fabrication, limiting adaptation to changing tissue environments and minimally invasive delivery. 4D printing addresses this limitation by introducing time-dependent transformation. In SMP-based 4D printing, the scaffold is printed in a permanent geometry, programmed into a temporary shape, and recovered after implantation [28,57]. This reconciles two otherwise conflicting requirements: compact geometry for delivery and complex three-dimensional architecture for regeneration.

### 6.2. Patient-Specific Scaffold Design Using Anatomical Geometry

Patient-specific defects often have irregular shapes that standard scaffold designs cannot effectively treat. Imaging data from computed tomography (CT) and magnetic resonance imaging (MRI) can be used to reconstruct defect geometry and guide scaffold design [58]. When combined with SMPs, the scaffold can be engineered to match the target defect after shape recovery rather than only at the time of fabrication, which is particularly valuable for craniofacial defects, irregular bone voids, osteochondral lesions, vascular structures, and soft-tissue reconstruction. A patient-specific SMP scaffold may be fabricated in a final-defect geometry but temporarily programmed into a smaller or simpler configuration for implantation, recovering into a structure that improves defect filling, tissue contact, and mechanical stability while reducing the need for extensive intra-operative shaping.

### 6.3. Spatial Control of Porosity, Stiffness, and Deformation

A major advantage of 4D printing is spatial control of scaffold properties. Different regions may require different pore structures, stiffness values, degradation rates, or deformation behaviors. An osteochondral scaffold, for instance, requires a stiff mineralized region for bone integration and a softer region for cartilage repair; vascular or neural scaffolds may require anisotropic channels that guide cell migration or tissue alignment [30,59]. SMP-based 4D printing supports such spatial programming through controlled material distribution, printing pattern, fiber orientation, and local architecture, enabling folding structures, expanding scaffolds, self-opening channels, and curvature-matched implants. 4D printing therefore offers a route to scaffolds that are not only patient-specific in shape but also functionally heterogeneous.

### 6.4. Multi-Material Printing for Heterogeneous Tissue Interfaces

Many regenerative targets involve interfaces between tissues with different mechanical and biological properties, including bone-cartilage, tendon-bone, muscle-tendon, vascular-soft tissue, and neural interfaces. A single homogeneous material rarely reproduces these gradients. Multi-material 4D printing combines SMPs with bioactive, conductive, degradable, or mechanically distinct components, so that different scaffold regions can be assigned different functions [28,57]. Yet multi-material printing also raises new concerns. Interfacial bonding, mismatched thermal behavior, differential degradation, and printing compatibility must be carefully controlled to avoid structural failure or unpredictable shape recovery.

### 6.5. Manufacturing Challenges in Resolution, Reproducibility, and Scale-Up

Despite its potential, 4D printing of SMPs faces several manufacturing hurdles. Shape memory behavior is sensitive to printing temperature, extrusion pressure, curing conditions, layer orientation, cooling rate, and post-processing; small parameter variations can alter crystallinity, crosslinking density, porosity, *T_trans_*, and recovery behavior. Reproducibility is therefore a critical issue for both research and clinical translation [60].

Resolution is another limitation. Tissue engineering scaffolds often demand micro- to mesoscale features for cell behavior and vascularization, while many SMP printing methods are limited in feature size, material choice, or printing speed. Patient-specific fabrication must also be compatible with standardized quality control and regulatory requirements. Scale-up imposes the additional challenge of preserving programmed temporary shapes during packaging, storage, transport, and surgical handling. If the temporary geometry relaxes before implantation, scaffold function may be lost. Manufacturing workflows should therefore be co-designed with programming, sterilization, and packaging strategies, treating 4D printing as part of a complete regenerative-device engineering process rather than an isolated fabrication step.

Overall, 4D printing provides a key bridge between SMP design and clinically relevant regenerative architectures. Its impact will ultimately depend on whether printed constructs can be manufactured reproducibly, activated safely, and validated under biologically and clinically relevant conditions.

## 7. Applications in Tissue Engineering and Regenerative Medical Devices

**Figure 5:**
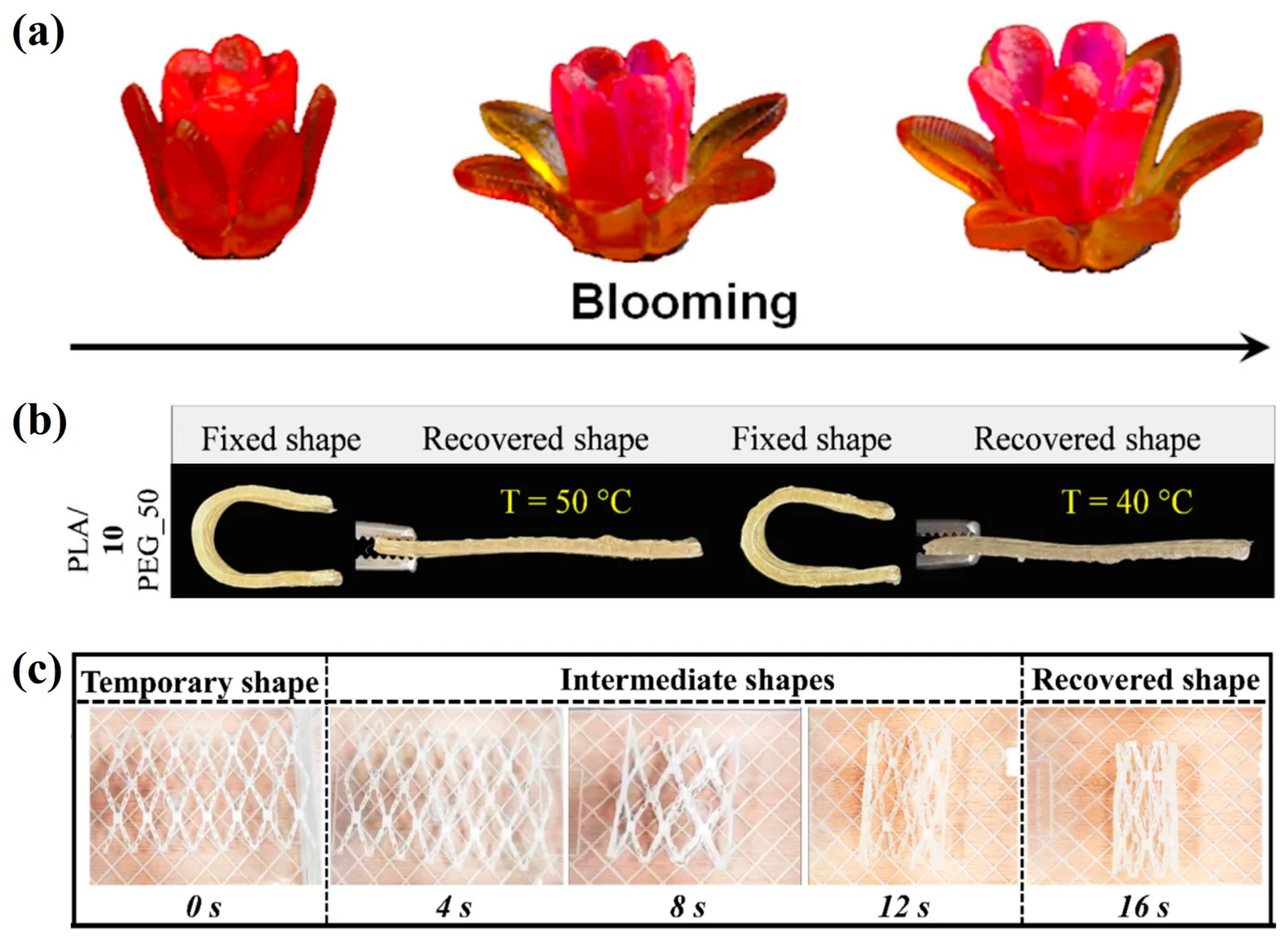
Representative real and additively manufactured SMP constructs for regenerative applications. (**a**) Multimaterial 4D-printed SMP architecture exhibiting programmed, tailorable shape transformation. (**b**) 3D-printed PLA/PEG gyroid scaffold shown in its fixed (temporary) and recovered (permanent) shapes after thermal activation near body temperature (recovery ratio of approximately 97% at 40 °C). (**c**) Fused-deposition-modeled PLA SMP porous vascular scaffold designed for self-expansion (recovery ratio of approximately 98% at 47 °C). Panel (**a**) is reproduced from ref. [28], and panels (**b**,**c**) from refs. [22,61], all under the CC BY 4.0 license.

SMPs have been explored across diverse tissue engineering applications because they enable functions difficult to achieve with conventional static scaffolds. Programmable deformation, recoverable predefined geometry, and adaptation to confined or irregular defect environments are particularly valuable in regenerative settings. The following sections summarize representative applications, emphasizing how SMP-enabled functions contribute to tissue repair rather than enumerating material systems. Representative real and additively manufactured SMP constructs, spanning a 4D-printed multimaterial architecture and 3D-printed bone and vascular scaffolds, illustrate fabricated examples relevant to these applications (Figure 5). Tissue-specific design considerations are summarized (Table 3).

### 7.1. Bone and Osteochondral Regeneration

**Figure 6:**
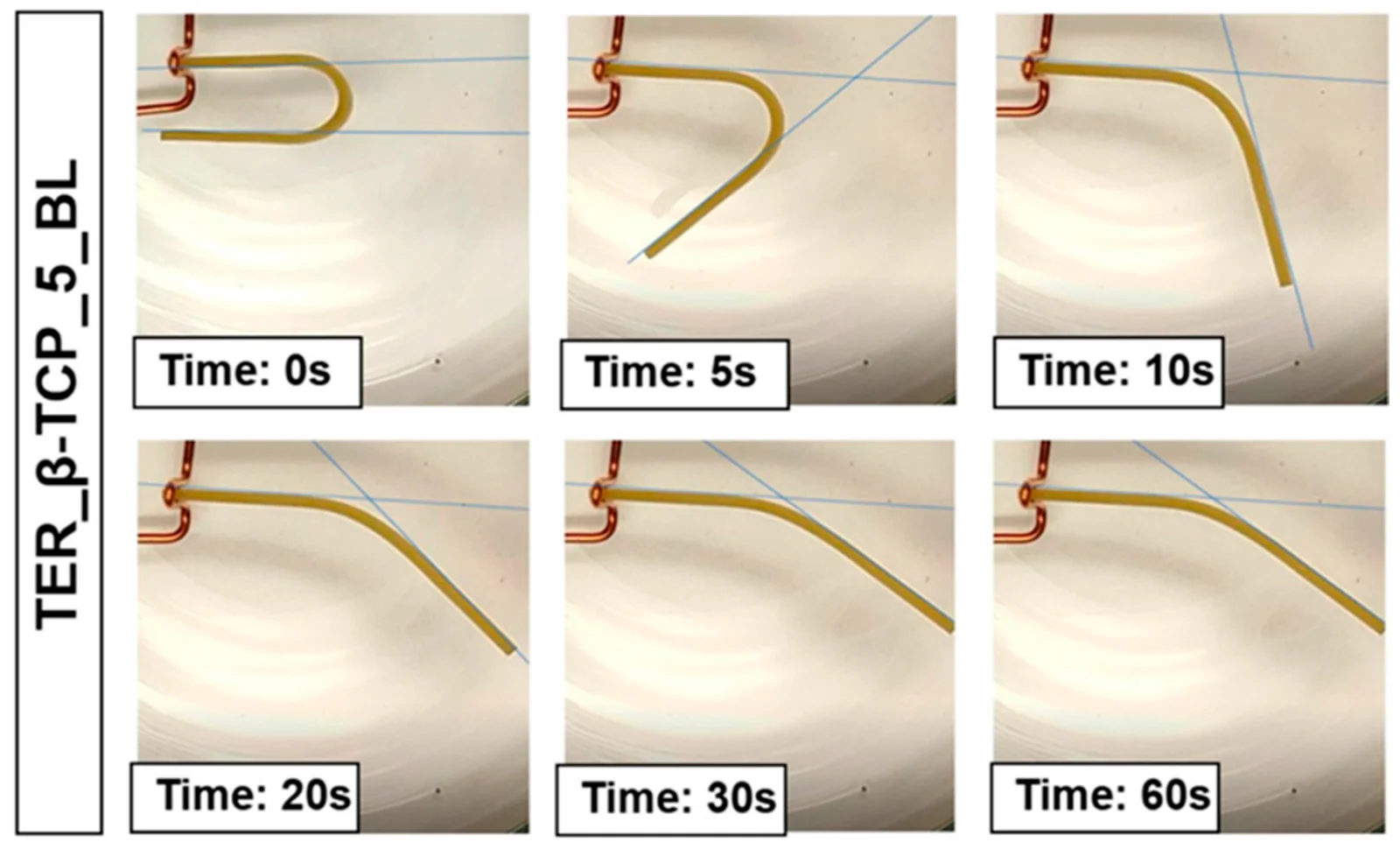
3D-printed shape memory terpolymer scaffold material reinforced with 5 wt% β-tricalcium phosphate (β-TCP), shown recovering from a programmed U-shaped temporary form toward its original shape upon thermal activation over 60 s (recovery ratio above 80% at body temperature). Adapted from ref. [23] under the CC BY 4.0 license.

Bone tissue engineering frequently requires scaffolds that can fill irregular defects, provide temporary mechanical support, and promote osteogenic tissue formation. Conventional prefabricated scaffolds often fail to match complex defect geometries, leading to poor host-tissue contact, unstable fixation, or uneven bone ingrowth. SMP-based scaffolds address these limitations by being inserted in compact form and recovering into a defect-filling architecture (Figure 6) [40,53]. Polyester-based SMPs and composite SMPs incorporating HA, CaP, or bioactive glass are particularly relevant because they combine shape recovery with osteoconductivity and improved stiffness [49,62]. In osteochondral applications, region-specific design can simultaneously support subchondral bone repair and cartilage regeneration. Bone-relevant SMP scaffolds must, however, balance *F_rec_* and mechanical strength against degradation behavior, since excessive stiffness or slow degradation can interfere with remodeling, while insufficient mechanical stability may lead to scaffold collapse before new tissue forms.

### 7.2. Cartilage and Musculoskeletal Soft-Tissue Repair

Cartilage, tendon, ligament, and other musculoskeletal soft tissues require scaffolds that withstand mechanical loading while retaining flexibility. Shape memory behavior supports minimally invasive implantation, defect conformity, and dynamic mechanical conditioning. An SMP scaffold can be programmed into a compact shape for insertion and recover to match the defect site or restore a load-bearing porous structure [39,54]. Cartilage and soft-tissue applications generally favor more compliant systems, including SMPUs, hydrogel-like SMPs, and elastomeric biodegradable networks. SMP scaffolds can also influence cell differentiation through changes in stiffness, strain distribution, or scaffold architecture. For example, a body-temperature-triggered ternary shape-memory scaffold carrying an immobilized chondrogenic agent enabled minimally invasive delivery and supported cell-free cartilage regeneration in vivo [63]. The dominant challenge is maintaining mechanical function under repetitive loading without imposing excessive stress on regenerating tissue, making long-term fatigue resistance and stable integration essential design considerations.

### 7.3. Vascular and Endoluminal Regenerative Devices

Vascular and endoluminal tissues impose unique design constraints because regenerative devices must be delivered through narrow lumens, recover within confined spaces, and maintain contact with curved or tubular tissues. SMPs are well suited to these requirements because they can be temporarily compressed and later expanded into tubular, porous, or conformal structures [37,38]. SMP-based vascular grafts, conduits, and endoluminal scaffolds may improve deployment, luminal contact, and anatomical fit, while their lower stiffness compared with metallic systems can reduce mechanical mismatch with soft vascular tissues. Conductive or bioactive SMP composites may further promote endothelialization, guide tissue integration, or deliver therapeutic molecules. Vascular applications, however, demand strict control of *F_rec_*, surface thrombogenicity, mechanical fatigue, and dimensional stability. The scaffold must expand reliably without excessive pressure on the vessel wall and maintain patency under dynamic flow conditions.

### 7.4. Cardiac and Neural Tissue Engineering

Cardiac and neural tissues are highly sensitive to mechanical and electrical cues, making them important targets for functional SMP-based scaffolds. In cardiac repair, SMP patches can provide conformal contact with the beating heart, support mechanical integration, and incorporate conductive components for electrical coupling; their shape recovery can adapt a patch to curved myocardial surfaces or enable less invasive delivery [7,50]. For neural tissue engineering, SMP-based conduits and scaffolds may guide nerve regeneration by recovering into aligned or channel-containing structures. For example, an electrospun shape memory nanofibrous mat can be loaded with cells in a flat temporary configuration and then self-form into a multichannel conduit at body temperature, improving the repair of long nerve gaps [64]. Soft and flexible SMP systems are essential because excessive stiffness can damage neural tissue and impair regeneration. Conductive fillers or bioactive molecules can further enhance neural guidance and signal transmission. Both cardiac and neural applications, however, demand gentle actuation, high cytocompatibility, and minimal inflammatory response, and activation methods must be carefully selected because thermal or photothermal stimulation can be harmful to sensitive tissues.

### 7.5. Skin, Wound Healing, and Soft-Tissue Reconstruction

Skin and wound-healing applications benefit from scaffolds or dressings that conform to irregular wound beds, maintain close contact with tissue, and support cell migration and matrix formation. SMP-based wound matrices can be programmed for easy handling and recover to improve coverage of complex wound surfaces, which is useful for deep wounds, burns, diabetic ulcers, and irregular soft-tissue defects [65]. Hydrogel-like SMPs, SMPUs, and natural-polymer composites are particularly relevant because they combine flexibility, moisture retention, and biological functionality. SMP wound systems may further incorporate antimicrobial agents, growth factors, or drug-delivery components. The principal design challenge is achieving sufficient shape recovery and tissue contact while maintaining softness, breathability, and compatibility with the wound microenvironment, with inflammation control and degradation-product safety being especially critical for chronic wounds.

Across bone, cartilage, vascular, cardiac, neural, and wound-healing applications, the central advantage of SMPs is their ability to connect scaffold structure with regenerative function through programmable transformation. Each tissue, however, imposes distinct requirements for stiffness, *F_rec_*, degradation, activation safety, and host response, so successful application demands tissue-specific engineering rather than universal scaffold design. Representative cytocompatibility and biological evaluations of SMP systems reported in the literature, including the cell type or model, the principal biological result, and the associated limitation, are summarized for comparison (Table 4).

## 8. Translational Barriers and Design Considerations

**Figure 7:**
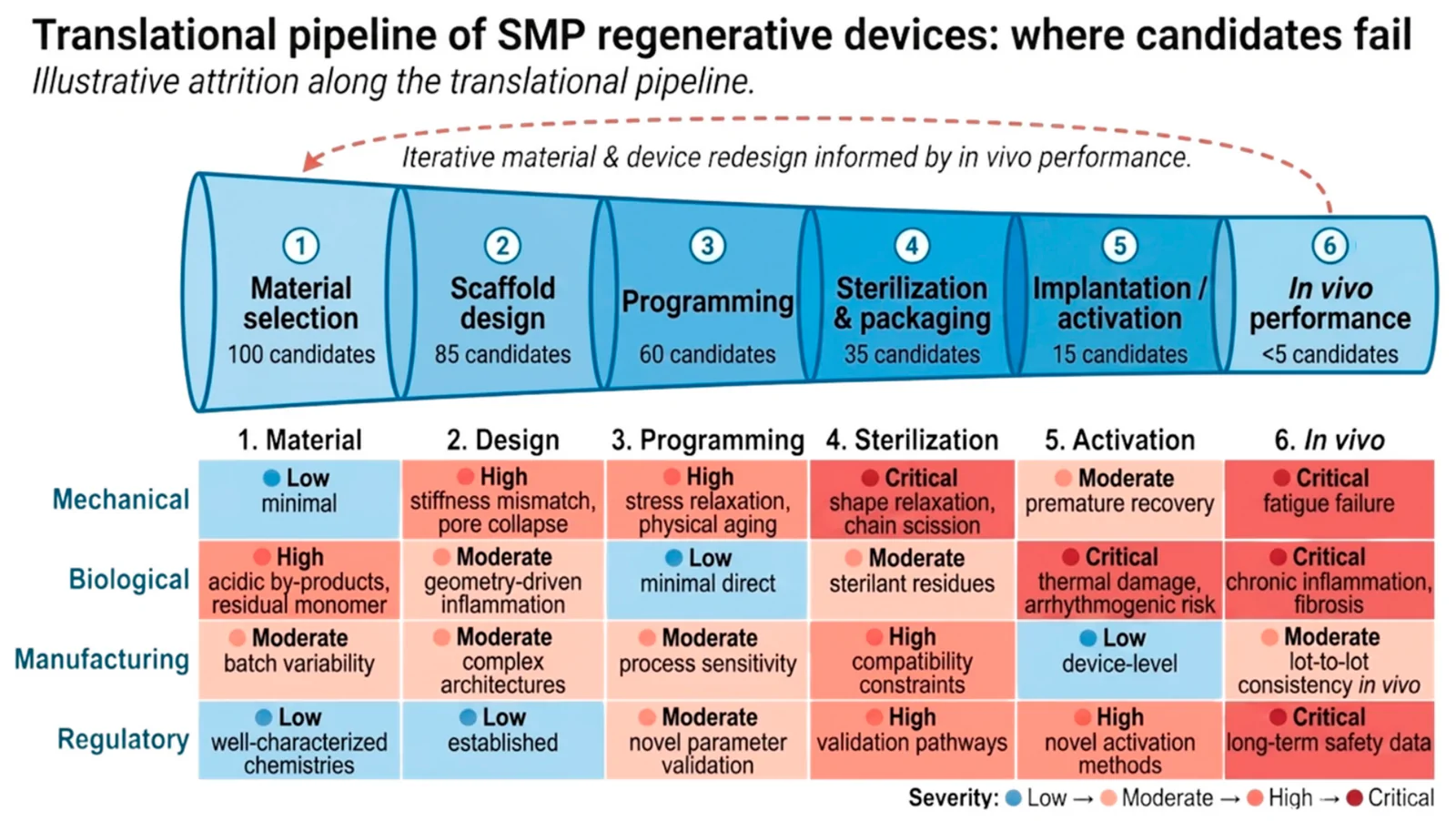
Translational pipeline of SMP-based regenerative medical devices and the distribution of critical risks across stages. The horizontal funnel illustrates progressive attrition of candidate devices through six translational stages, from material selection (**1**) to in vivo performance (**6**), with stage 6 highlighted in coral as the in vivo validation endpoint. A dashed arrow indicates iterative material and device redesign informed by in vivo outcomes. The aligned heatmap below maps risk severity (Low/Moderate/High/Critical) across four risk categories (Mechanical, Biological, Manufacturing, Regulatory) for each stage, identifying where translational risk concentrates. Candidate counts shown are illustrative and convey relative attrition along the pipeline; they do not represent published statistics.

While SMPs offer attractive functions for dynamic tissue engineering, their clinical translation remains challenging. Many SMP-based scaffolds show promising shape recovery, biocompatibility, or regenerative potential in laboratory studies, but medical-device development requires additional validation under practical conditions. For clinical use, SMP scaffolds must maintain programmed shape, recover safely, perform mechanically, degrade predictably, and remain biologically compatible after manufacturing, sterilization, packaging, storage, and implantation [60,67]. Translational success therefore depends not only on material innovation but also on process stability, device reliability, and regulatory readiness (Figure 7).

### 8.1. Sterilization-Induced Changes in Shape Memory Behavior

Sterilization is essential for implantable devices but can substantially alter SMP behavior. Thermal sterilization may induce partial shape recovery, stress relaxation, or changes in crystallinity when *T_trans_* is near the sterilization condition [68]. Radiation-based methods such as gamma irradiation and electron beam can cause chain scission, additional crosslinking, or oxidation, shifting *T_trans_*, modulus, degradation rate, and recovery behavior. Chemical sterilization (for example, ethylene oxide) can be less thermally aggressive, but residual sterilant and possible chemical interactions must be considered. Because SMP-based scaffolds often need to retain a programmed temporary shape, sterilization should not be treated as a final processing step. Sterilization compatibility should be evaluated early in scaffold development, alongside *R_f_*, *R_r_*, mechanical performance, and biological safety.

### 8.2. Packaging and Storage of Programmed Structures

Unlike conventional scaffolds, SMP-based devices may be stored in temporary programmed configurations, raising additional shelf-life concerns. Physical aging, stress relaxation, hydration, plasticization, or thermal fluctuation can reduce *R_f_* and alter recovery behavior over time. Packaging must protect both the material and the programmed geometry. Storage temperature for thermally activated SMPs should remain well below *T_trans_*; water-responsive systems require humidity control, and biodegradable SMPs require minimal hydrolytic degradation during storage. SMP-based scaffolds therefore demand device-specific packaging strategies rather than standard scaffold storage conditions.

### 8.3. Long-Term Biocompatibility and Degradation-Product Safety

Biocompatibility is often evaluated through short-term cytotoxicity assays, but regenerative devices require longer-term assessment. SMP scaffolds interact with tissues during inflammation, cell infiltration, vascularization, matrix deposition, and remodeling, so biological response should be evaluated over time and under conditions reflecting the target environment [18,44]. For biodegradable SMPs, degradation products are a major translational consideration. Scaffolds must degrade at rates compatible with tissue formation while maintaining mechanical integrity early in regeneration. Degradation products must not induce local acidity, cytotoxicity, chronic inflammation, or impaired remodeling. Composite SMPs require additional evaluation because fillers, nanoparticles, ceramics, conductive materials, or bioactive agents can alter degradation behavior and host response. Long-term biocompatibility should therefore include macrophage response, fibrosis, vascularization, integration, and degradation-product safety, not cell viability alone.

### 8.4. Mechanical Fatigue and In Vivo Reliability

SMP-based regenerative devices are typically deployed in mechanically active environments. Bone, cartilage, tendon, cardiac, vascular, and soft tissues all impose distinct loading patterns, including compression, tension, bending, pulsation, and cyclic deformation. A scaffold that performs well in a single recovery test may fail under repeated loading or prolonged physiological exposure. Mechanical fatigue can change scaffold stiffness, pore structure, recovery behavior, and tissue contact, and repeated deformation in dynamic tissues may also accelerate degradation or cause microcracking, delamination, filler release, or loss of mechanical integrity [69]. Mechanical testing should therefore extend beyond initial modulus and *R_r_* to include fatigue resistance, creep, stress relaxation, cyclic recovery, and mechanical retention during degradation, evaluated according to the target tissue and expected implantation period.

### 8.5. Manufacturing Reproducibility and Quality Control

Shape memory behavior is highly sensitive to manufacturing conditions. Polymer composition, crosslinking density, crystallinity, phase separation, filler dispersion, printing parameters, cooling rate, and post-processing all influence *T_trans_*, *R_f_*, *F_rec_*, and degradation [60]. This sensitivity is amplified in porous scaffolds, multi-material systems, and patient-specific 4D-printed devices. Clinical translation requires reproducibility at both material and device levels: each scaffold should display consistent geometry, porosity, mechanical performance, recovery behavior, and biological safety. Patient-specific manufacturing introduces an additional challenge because geometry varies but functional performance must remain predictable. Quality control should therefore include not only chemical and mechanical characterization but also shape-memory-specific metrics such as programmed-shape stability, recovery accuracy, activation window, and deployment reliability.

### 8.6. Regulatory Validation of Shape-Changing Regenerative Devices

Regulatory evaluation of SMP-based regenerative devices is challenging because these systems combine features of smart materials, implantable scaffolds, biodegradable devices, and sometimes drug-delivery, bioactive fillers, or electronic components. Their performance cannot be fully captured by conventional static-scaffold tests. Regulators may require evidence that the device maintains its intended shape before implantation, activates safely, recovers predictably, remains mechanically reliable, and degrades without harmful effects [70]. The pathway becomes more complex when scaffolds are patient-specific, 4D-printed, cell-laden, bioactive, or remotely activated. In such cases, validation should encompass material biocompatibility, manufacturing consistency, sterilization stability, activation safety, degradation profile, and device-specific function. Early consideration of regulatory requirements can reduce late-stage risk and guide selection of clinically realistic materials, activation methods, and scaffold designs.

Translation of SMP-based tissue engineering platforms therefore requires a shift from proof-of-concept shape recovery to full device validation. Sterilization, packaging, storage, degradation, fatigue, manufacturing, and regulatory issues should be integrated into the design process from the outset, not deferred. This translational perspective is essential for developing SMP scaffolds that are scientifically innovative, clinically reliable, manufacturable, and suitable for regenerative medical-device applications.

## 9. Future Perspectives

Future progress will depend on moving beyond simple demonstrations of shape recovery toward systems that are biologically functional, mechanically reliable, manufacturable, and clinically translatable. Several directions appear particularly important:

**Tissue-specific design frameworks, not universal scaffolds.** Standardized, application-specific checklists linking *T_trans_*, *R_f_*, *R_r_*, *F_rec_*, degradation rate, and biological response to defined tissue targets (stiff, osteoconductive designs for bone; gentle, compliant, low-inflammation designs for cardiac, neural, and soft tissue) would unify currently fragmented evaluation.

**Integration with mechanobiology-guided design.** Since regeneration responds to stiffness, strain, stress relaxation, and dynamic deformation, future work should establish how programmed shape recovery influences cell behavior, ECM deposition, vascularization, and tissue maturation in vivo.

**Convergence with 4D printing, computational modeling, and artificial intelligence.** Combining spatial 4D-printing control with predictive modeling and machine-learning optimization of compositions, architectures, and activation conditions could reduce trial-and-error and enable personalized device design.

**Multifunctional SMP systems.** Integrating bioactive, immunomodulatory, conductive, sensing, drug-delivery, or remote-actuation functions could unite reconstruction with monitoring, therapeutic release, or stimulation, but each added component affects degradation, biocompatibility, sterilization, mechanics, and regulatory classification, so translational feasibility must be considered from the outset.

**Standardization and validation for clinical translation.** Application-specific protocols that test sterilization stability, programmed-shape shelf life, mechanical fatigue, degradation-product safety, and manufacturing reproducibility, and that connect material properties to device performance, are essential, especially for 4D-printed and patient-specific scaffolds.

With these advances, SMPs are well positioned to evolve from emerging smart polymers into clinically validated functional platforms for next-generation tissue engineering and regenerative medicine.

## 10. Conclusions

Shape memory polymers represent a versatile class of functional polymers whose programmable shape transformation directly addresses several long-standing challenges in tissue engineering. Their ability to be delivered in compact temporary configurations, recover predefined three-dimensional architectures, and adapt to defect-specific environments enables minimally invasive deployment, self-fitting behavior, dynamic mechanical conditioning, and staged tissue remodeling. However, regenerative performance is governed by the integrated control of polymer chemistry, *T_trans_*, *F_rec_*, *R_f_*, degradation behavior, and biological response, and not by shape recovery alone.

This review has emphasized that SMP-based regenerative platforms should be evaluated as engineered systems rather than smart materials in isolation. Each material platform, including polyester-based biodegradable SMPs, segmented polyurethanes, hydrogel-like networks, natural-polymer composites, and dynamic covalent systems, offers distinct trade-offs that must be matched to tissue-specific demands across bone, cartilage, vascular, cardiac, neural, and wound applications. Programmed scaffold functions and 4D-printed architectures further extend the design space, enabling patient-specific geometry and spatially heterogeneous performance. Yet translational success will hinge on factors that are too often considered downstream, including sterilization stability, programmed-shape shelf life, mechanical fatigue, manufacturing reproducibility, and regulatory readiness. Integrating these constraints into early-stage design is essential if SMPs are to evolve from demonstrative smart materials into clinically reliable regenerative devices.

## Figures and Tables

**Table 1:** Representative mechanical and shape-memory properties of SMP systems reported in the literature for tissue engineering and regenerative devices, with the corresponding measurement methods.

SMP System (Target)	Transition Temperature	Mechanical Properties	Shape Recovery	Measurement Method	Refs.
Crosslinked PCL self-fitting scaffold (bone defect filling; self-expanding stent); linear-PCL-diacrylate (DA) and star-PCL-TA	*T_m_* ≈ 55 °C (linear-PCL-DA), tunable to ≈37 to 46 °C via star-PCL-TA architecture and reduced molecular weight	Compressive modulus ≈5 to 7 MPa (linear-PCL-DA), decreasing to ≈0.7 to 2 MPa for lower-crystallinity star-PCL-TA; compressive strength ≈28 to 33 MPa (linear) versus ≈5 to 11 MPa (star); porosity ≈65 to 77%	*R_f_* and *R_r_* ≈ 100% (two cycles)	DSC (*T_m_*); static compression (modulus, strength at 85% strain); model-defect press-fit shape fixity and recovery	[40,41]
3D-printed PLA/poly(ethylene glycol) (PEG) gyroid scaffold (bone)	*T_g_* ≈ 36 °C (PLA *T_g_* ≈ 55 to 65 °C, lowered by 10 wt% PEG plasticizer)	Not reported (study focused on thermal transitions, crystallinity, and shape recovery)	*R_r_* ≈ 97% at 40 °C (within ≈ 6 min)	DSC (*T_g_*); shape-recovery (bending) test; ATR-FTIR, Raman, XRD, SEM	[29]
FDM PLA porous vascular scaffold (vascular)	*T_g_* = 42.15 °C	Ultimate tensile strength 29.5 MPa at room temperature, decreasing to 11.6 MPa at elevated temperature; maximum tensile modulus ≈ 34 MPa at room temperature	*R_r_* ≈ 98%	DMA (*T_g_*, storage modulus); uniaxial tensile test (strength, modulus); DSC; TGA; SEM	[30]
Water-based polyurethane with superparamagnetic iron oxide (SPIO) nanoparticles, 3D-printed bone scaffold	Thermo- and water-responsive	Not reported here (printability and osteogenic induction emphasized)	Confirmed by bending method (in air and water)	Low-temperature fused deposition manufacturing (3D printing); shape-recovery (bending) test	[42]

Abbreviations: TA, triacrylate; DSC, differential scanning calorimetry; DMA, dynamic mechanical analysis; TGA, thermogravimetric analysis; SEM, scanning electron microscopy; XRD, X-ray diffraction; ATR-FTIR, attenuated total reflectance Fourier-transform infrared spectroscopy; FDM, fused deposition modeling.

**Table 2:** Comparison of representative SMP material platforms relevant to tissue engineering and regenerative medical devices.

SMP Platform	Representative Materials	Transition/Activation	Key Strengths	Limitations/Concerns
Polyester-based (semi-crystalline)	PCL, PLA, PLGA, PCL/PLA and PCL/PEG copolymers, PLLA-TMC networks	*T_m_*-based; ~37 to 60 °C	Biodegradability; processability; bone-relevant stiffness; well-established cytocompatibility	Acidic by-products (PLA/PLGA); slow PCL degradation; modest *F_rec_*
Polyurethane-based (segmented)	Aromatic/aliphatic SMPUs; PCL-diol or PEG soft segments; lysine-derived SMPUs	*T_g_*- or *T_m_*-based; 30 to 80 °C	Tunable elasticity; high *R_r_*; soft-tissue mechanical match; large strain	Isocyanate residues; slow or variable degradation; possible chronic inflammation
Hydrogel-like/water-responsive	PEG networks, PVA, gelatin/PEG composites, supramolecular gels	Hydration- or temperature-triggered	Cell compatibility; ECM-like environment; mild activation; cell-laden compatible	Low strength; rapid swelling; limited load-bearing use
Natural-polymer/bioactive composites	Collagen-, gelatin-, silk-, chitosan-blended SMPs; hydroxyapatite (HA)/Calcium phosphate (CaP)/bioglass-loaded SMPs	Polymer-class dependent	Cell adhesion; osteoconductivity; tunable degradation; bioactive cues	Variability of natural components; filler dispersion; altered *T_trans_*
Stimuli-responsive composites (remote)	Fe_3_O_4_-, gold nanoparticles, polypyrrole (PPy)-loaded SMPs	Magnetic, NIR-photothermal, electrical	Spatial or on-demand actuation; multi-stimuli capability; bioelectronics integration	Heating safety; nanoparticle leaching; long-term clearance unclear
Dynamic covalent/vitrimer-like	Vitrimers based on transesterification, imine, disulfide bonds	Bond-exchange	Reprocessability; self-healing; reprogrammable scaffolds	Stability under physiological conditions; chemical safety; early biomedical stage

Abbreviations: SMPU, shape memory polyurethane; ECM, extracellular matrix; NIR, near-infrared.

**Table 3:** Tissue-specific design requirements and critical risks for SMP-based regenerative devices.

Target Tissue	Mechanical Demand	Preferred SMP Class	Key Functional Requirement	Activation Mode	Critical Risk
Bone/osteochondral	Stiff, load-bearing (E ≈ 100 MPa to 10 GPa)	Polyester or polyester/HA composite SMPs	Self-fitting in irregular voids; osteoconductivity	Body T or warmed saline	Premature degradation; insufficient *F_rec_*
Cartilage/soft musculoskeletal	Compliant, viscoelastic, fatigue-resistant	Polyurethane SMPs; reinforced hydrogel SMPs	Compliance matching; cyclic recovery	Body T	Mechanical fatigue; pore loss under loading
Vascular/endoluminal	Highly elastic, pulsatile	SMPU; polyester-PU blends	Self-expanding lumen; controlled radial force	Body T	Thrombogenicity; over-expansion
Cardiac patches	Soft, anisotropic, dynamic	Conductive SMP composites; SMPU	Conformal contact; possible electrical coupling	Body T; magnetic	Local heating; arrhythmogenic risk
Neural conduits	Very soft, channel-aligned	Hydrogel-like SMPs; soft PCL networks	Aligned microchannels; gentle actuation	Body T; light	Photothermal damage; chronic inflammation
Skin/wound healing	Compliant, breathable	Hydrogel-like SMPs; natural-polymer composites	Wound-bed conformity; antimicrobial or drug delivery	Body T; moisture	Premature softening; degradation byproducts

**Table 4:** Representative cytocompatibility and biological evaluations of SMP systems reported in the literature, summarizing the cell type or model, the principal biological result, and the main limitation of each study.

SMP System (Target)	Cell Type/Model	Biological Result	Limitation	Ref.
Polydopamine-coated PCL-DA self-fitting scaffold (bone)	Osteoblasts (in vitro)	Improved osteoblast adhesion, proliferation, and osteogenic gene expression; apatite formation vs uncoated	In vitro; high melt transition (≈55 °C)	[40]
3D-printed water-based PU with SPIO (bone)	Human mesenchymal stem cells (hMSCs) (in vitro)	Enhanced hMSC osteogenesis via SPIO release (≈2.7× for PU/PEO)	In vitro; SPIO-dependent; modest strength	[42]
Electrospun shape-memory scaffold (bone/osteogenic)	Human adipose-derived stem cells (hASCs) (in vitro)	Osteogenic capacity preserved after shape-memory triggering	In vitro; preserved, not enhanced	[66]
Electrospun shape-memory PU nanofibers (cell conditioning)	hASCs (in vitro)	Fiber-alignment switch reoriented viable cells and directed cell morphology	In vitro; hyperthermic actuation	[54]
Self-deploying SMP graft and sleeve(segmental bone defect)	In vivo (mouse femoral defect)	Integration and defect stability at 12 weeks (torsion comparable to allograft); no gross new bone	No de novo bone formation; small-animal model	[53]
Polyhedral oligomeric silsesquioxane (POSS)-poly(D,L-lactide) nanocomposite (biodegradable)	In vivo (rat subcutaneous)	Mild foreign-body response; inflammation increased with faster (shorter-PLA-arm) degradation	Stronger inflammation with faster degradation; subcutaneous model	[62]

## Data Availability

No new data were created or analyzed in this study. Data sharing is not applicable.
